# 1-Kestose Blocks UVB-Induced Skin Inflammation and Promotes Type I Procollagen Synthesis via Regulating MAPK/AP-1, NF-κB and TGF-β/Smad Pathway

**DOI:** 10.4014/jmb.2311.11020

**Published:** 2024-02-22

**Authors:** Jihye Baek, Jong-Hwa Kim, Jiwon Park, Do Hyun Kim, Soonok Sa, Jung-Sook Han, Wonyong Kim

**Affiliations:** 1 Department of Microbiology, Chung-Ang University College of Medicine, Seoul 06974, Republic of Korea; 2 Food R&D Center, Samyang Corp., Seongnam 13488, Republic of Korea

**Keywords:** 1-Kestose, HaCaT cells, UVB, anti-photoaging, anti-inflammatory, MAPK/AP-1, NF-κB, TGF-β/Smad

## Abstract

Solar UVB irradiation cause skin photoaging by inducing the high expression of matrix metalloproteinase (MMPs) to inhibit the expression of Type1 procollagen synthesis. 1-Kestose, a natural trisaccharide, has been indicated to show a cytoprotective role in UVB radiation-induced-HaCaT cells. However, few studies have confirmed the anti-aging effects. In the present study, we evaluated the anti-photoaging and pathological mechanism of 1-kestose using Human keratinocytes (HaCaT) cells. The results found that 1-kestose pretreatment remarkably reduced UVB-generated reactive oxygen species (ROS) accumulation in HaCaT cells. 1-Kestose suppressed UVB radiation-induced MMPs expressions by blocking MAPK/AP-1 and NF-κB p65 translocation. 1-Kestose pretreatment increased Type 1 procollagen gene expression levels by activating TGF-β/Smad signaling pathway. Taken together, our results demonstrate that 1-kestose may serve as a potent natural trisaccharide for inflammation and photoaging prevention.

## Introduction

With the global trend of population aging and the improvement of living standards worldwide, aging and aging-related diseases have become more prominent [1]. Two types are responsible for skin aging such as extrinsic aging (photoaging) and intrinsic aging. Intrinsic aging occurs naturally in the skin, and once it progresses, the barrier function against ultraviolet (UV) radiation is damaged, making the skin more susceptible to photoaging. UV radiation from the sun is an extrinsic aging factor contributing to skin aging, with particular exposure to the UVB radiation (280–315 nm) spectrum. Chronic exposure to UVB radiation can lead to skin aging characterized by increased skin vulnerability, laxity, roughness, dryness, hyperpigmentation, blister formation, and wrinkle formation [2].

Excessive exposure to UVB can lead to an imbalance in cellular redox status and promote the accumulation of reactive oxygen species (ROS), which are known to trigger inflammatory mediators [3]. UVB-generated ROS are a serious threat to biomembranes and induce the high expression of matrix metalloproteinase (MMPs), which deteriorates collagen and elastin fibers in the dermal extracellular matrix (ECM) [4]. The mitogen-activated protein kinases (MAPKs), including c-Jun N-terminal kinase (JNK), extracellular signal-regulated kinase (ERK), and p38 MAPK (p38) are important signaling mediators that transmit extracellular signals to the nucleus [5]. UVB-generated ROS production stimulates the activation of MAPKs [6]. Afterwards, activator protein-1 (AP-1) and nuclear factor-κB (NF-κB) were activated by MAPK pathway, leading to the upregulation of MMPs secretion [7]. Secretion of MMPs in photoaged skin can be a major cause of reduced skin elasticity and wrinkle formation. Besides, the up-regulation of the MMPs pathway is a major target of UVB radiation-induced skin photoaging response [8]. Moreover, skin photoaging can amplify skin inflammation caused by UV irradiation. A previous study indicates that UV-induced cytokines of TNF-α, interleukin (IL)-6 and IL-8 could induce MMPs expression [9]. Thus, the expression of inflammatory cytokines through these signaling pathways can be a critical point in preventing UV-induced photoaging.

Beyond collagen degradation, UVB has been shown to influence the synthesis of pro-collagen. TGF-β/Smad signaling pathway is reported to be closely related to Type I procollagen synthesis. TGF-β specifically binds to cell surface receptors, initiating cellular responses, and then activates the Smad2/3 transcription factors [10, 11]. Subsequently, the signal was transmitted intracellularly, leading to an increase in Type I pro-collagen synthesis [12]. Therefore, the pro-collagen synthesis represents potential target for preventing photoaging.

1-Kestose (as a type of fructo-oligosaccharide) is a functional trisaccharide consisting of one sucrose with one fructose molecule and is naturally found in vegetables and fruits, such as onions, tomatoes, garlics and asparagus [13, 14]. Because of its low sweetness, low calorie, and the potential benefits of reducing the risk of type 2 diabetes, atopic dermatitis, and acute gastroenteritis, as well as its antioxidant effect, 1-kestose is considered a new food additive and widely applied in the food industry [15]. Previous studies have revealed that 1-kestose not only possesses various physiological functions, including anti-inflammatory properties, improvement of allergic conditions, and prevention of metabolic syndrome but also has anti-inflammatory, elastin, and moisturizing effects on the skin [16, 17]. These effects primarily occur from the role in controlling and regulating reactive oxygen species. However, the effects of 1-kestose in skin photoaging following UV radiation have not been fully investigated. Therefore, this study investigated the effects of 1-kestose on skin cellular responses, and photoaging under UVB radiation-induced oxidative damage was confirmed in vitro.

## Materials and Methods

### Reagents and Antibodies

1-Kestose powder was provided by Food R&D Center, Samyang Corp. (Republic of Korea). Radio-immunoprecipitation (RIPA) lysis buffer, TRIzol reagent, bovine serum albumin (BSA), 2'7'-dichlorofluorescein-diacetate (DCFH-DA), dimethyl sulfoxide (DMSO) and 4',6-diamidino-2-phenylindole (DAPI) were purchased from Sigma-Aldrich (USA). Fetal bovine serum (FBS), Dulbecco’s modified Eagle medium (DMEM) and penicillin/streptomycin were obtained from Gibco (BRL, USA). pGL3 basic vector and dual-luciferase reporter assay system (E1910) was purchased from Promega (USA). Protease inhibitor (#78425), lipofectamine 2000 reagent (Cat. 11668027) and PicoEPD reagent (EBP-1073) were purchased from Thermo Fisher Scientific (USA). Primary antibodies against MMP-1 (#54376), ERK (#4695), JNK (#9252), p38 (#8690), p-ERK (#4370), p-JNK (#4671), p-p38 (#4511), c-Fos, (#2250), c-Jun (#9165), p-c-Fos (#5348), p-c-Jun (#3270), TGF-β1 (#3711), Smad2/3 (#8685), p-Smad2/3 (#8828), and β-actin (#4970), as well as horseradish peroxidase (HRP) goat anti-rabbit IgG (#2895) were purchased from Cell Signaling Technology (USA). Human ELISA kit was obtained from R&D Systems (USA).

### Cell Culture and UVB Irradiation Treatment

The human keratinocyte cell line HaCaT cells were purchased from the Korean Cell Line Bank (Republic of Korea). Cells were cultured in a Dulbecco’s modified eagle media (DMEM, Lonza) containing 10% fetal bovine serum (FBS, Gibco) and 1% penicillin/streptomycin (Gibco). HaCaT cells were cultured at 37°C in a humidified incubator with 95% air condition and 5% CO_2_. Before UVB irradiation, and then immediately exposed to 40 mJ/cm^2^ of UVB radiation (312 nm, G15T8E, 15 W, Sankyo Denki, Japan). After UVB irradiation, the media was changed to serum-free media and continued to be cultured in the incubator for 24 h.

### Cytotoxicity Test

HaCaT cells were seeded in a 96 well plate at a density 10^4^ cells/well and cultured in the incubator for 24 h. Pretreated with 1-kestose and incubated at different concentrations in the range of 5–20 μM for 24 h. Then, the cells were UVB irradiation following the method in section Cell Culture and UVB Irradiation Treatment. Cell viability was detected by MTT colorimetric method [18]. Briefly, 200 μl DMEM media containing 0.5 mg/ml MTT reagent was added into each well and incubated at 37°C for 1 h. The supernatant was replaced with 100 μl of DMSO and absorbance was measured at 590 nm.

### Measurement of Reactive Oxygen Species (ROS)

HaCaT cells were seeded in 96 well plates irradiated with 40 mJ/cm^2^ UVB. The cells were treated with the 1-kestose for 12 h. The generation of ROS was measured by using DCFH-DA fluorescent probe. The intracellular ROS were visualized by a fluorescence microscope at the excitation and emission wavelengths of 485 and 535 nm, respectively.

### Gene expression Analysis

To quantify gene expression levels, the total RNA was isolated using the Trizol, and reversely transcribed using the Primescript first-strand cDNA synthesis kit. The cDNA was applied for quantitative RT-PCR, which was carried out in Quant3 real-time PCR system (Applied Biosystems, USA) with SYBR Green Master Mix. The relative expression levels were obtained with 2^-ΔΔCt^ method and β-actin acted as the control. The target gene primer sequences are shown in Table 1.

### Measurement of TNF-α, IL-6, and IL-8 Production

The culture supernatants of the treated HaCaT cells were obtained 12 h after irradiation and the level of TNF-a, IL-6 and IL-8 was detected using R&D system ELISA kits (R&D Systems) following the manufacturer’s instructions.

### MMP-1 Luciferase Reporter Assay

The luciferase reporter constructs of the pGL3-MMP-1 promoter generated as in a previous report [19]. HaCaT cells cultured in 24-well plates, and the medium was replaced fresh DMEM media. pGL-basic (0.5 ug) or pGL3-MMP-1 (0.5 ug) promoter encoding luciferase driven by the MMP-1 promoter was incubated with Lipofectamine 2000 reagent. Cells were treated with 1-kestose and irradiated with 40 mJ/cm^2^ UVB. After 12 h, cells were lysed and analyzed for dual-luciferase reporter activity. The relative amount of luciferase activity was designated to 1 after normalization to the *Renilla* luciferase signal in untreated cells.

### Western Blotting

The cells were lysated in RIPA buffer with protease inhibitor and phosphatase inhibitor. Cell lysates were separated by 12% SDS-PAGE and transferred to PVDF membranes. Then, the PVDF membranes were blocked with 3% BSA on a shaking table at room temperature for 1 h. Membranes were incubated with primary antibodies overnight. Membranes were washed with TBST 3 times, 10 min each time, added the corresponding secondary antibody with HRP-IgG at room temperature for 1 h. After washing 3 times with TBST, band intensity was analyzed by Image J software.

### Immunofluorescence

After irradiated by UVB, cells were treatment different concentration of 1-kestose for 12 h. Subsequently, cells were fixed with 4% paraformaldehyde at 37°C for 10 min, cells were the permeabilized by PBS containing 0.2%TritonX-100 for 15 min and blocked with 2% BSA for 1 h. NF-kB p65 antibodies containing 0.1% BSA was incubated at 4°C overnight and then incubated with green fluorescence dye (Alexa Flour 488 goat anti-rabbit IgG) for 1 h at room temperature. 500 ng/ml DAPI was used for unclear staining and cells were observed by fluorescence microscope (Olympus Opticals, Japan).

### Statistical Analysis

All the experiments were carried out in triplicates. Data were presented as means ± SD (*n* = 3) in figures. Statistical analyses were assessed by one-way ANOVA followed by Dunnett’s multiple comparison test (*p* < .05) for data comparisons using GraphPad software (GraphPad 8.0, USA).

## Results

### 1-Kestose Protect HaCaT Cells against UVB Radiation-Induced Damage

**Fig. 1:**
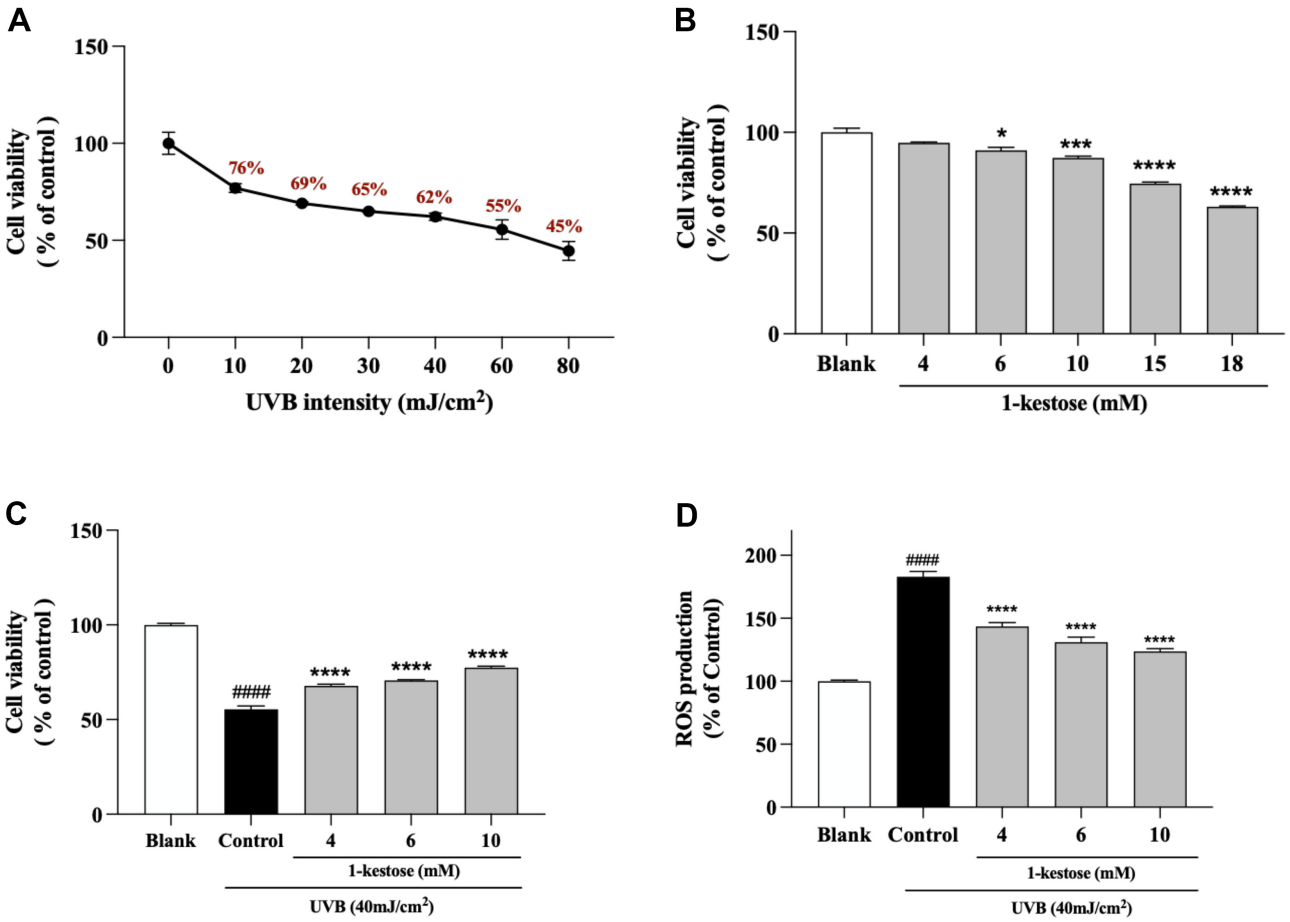
Effect of 1-kestose on cytotoxicity (MTT test) and ROS production in UVB radiation-induced human skin HaCaT cells. (**A**) Phototoxicity by various UVB dose (0, 10, 20, 30, 40, 50, and 80 mJ/cm^2^) (**B**) The viability of 1-kestose (4, 6, 10, 15, and 18 mM) was confirmed by MTT test of HaCaT cells for 24 h. (**C**) 1-kestose (4, 6, and 10 mM) combined with 40 mJ/cm^2^ UVB irradiation was identified on HaCaT cells for 24 h to confirm the protective effect by MTT test. (**D**) The effect of 1-kestose on intercellular reactive oxygen species (ROS) levels in UVB radiation-induced HaCaT cells. All data are expressed as mean ± SD (*n* = 3). #### *p* < .0001 vs. blank group, * *p* < .05, *** *p* < .001, and **** *p* < .0001 vs control.

As the UVB irradiation increased, cell viability significantly decreased. In this study, an appropriate UVB irradiation dose (40 mJ/cm^2^) was selected based on a significant difference (62%) compared with the blank group.(Fig. 1A). To evaluate the cytotoxic effect of 1-kestose, cells treated with various 1-kestose concentrations (4, 6, 10, 15, and 18 mM) for 24 h. Various 1-kestose concentrations (4, 6, and 10 mM) was no cytotoxicity on HaCaT cells but 15 mM and 18 mM were decreased to 25.6% and 36.9%, respectively (Fig. 1B). After UVB irradiation, viability decreased from 100% to 44.6%. When 1-kestose (4, 6, and 10 mM) treatment, cell viability showed 67.8%, 70.6%, and 77.2%, respectively (Fig. 1C).

Intracellular ROS generation of various concentrations of 1-kestose are shown in Fig. 1D. Control group (UVB radiation) was certainly indicating a high expression of DCFH-DA. 1-kestose inhibited ROS production with UVB irradiation. These results showed that 1-kestose (4, 6, and 10 mM) can effectively prevent UVB radiation-induced damaged HaCaT cells.

### 1-Kestose Attenuated UVB Radiation-Induced Pro-Inflammatory Secretion

**Fig. 2:**
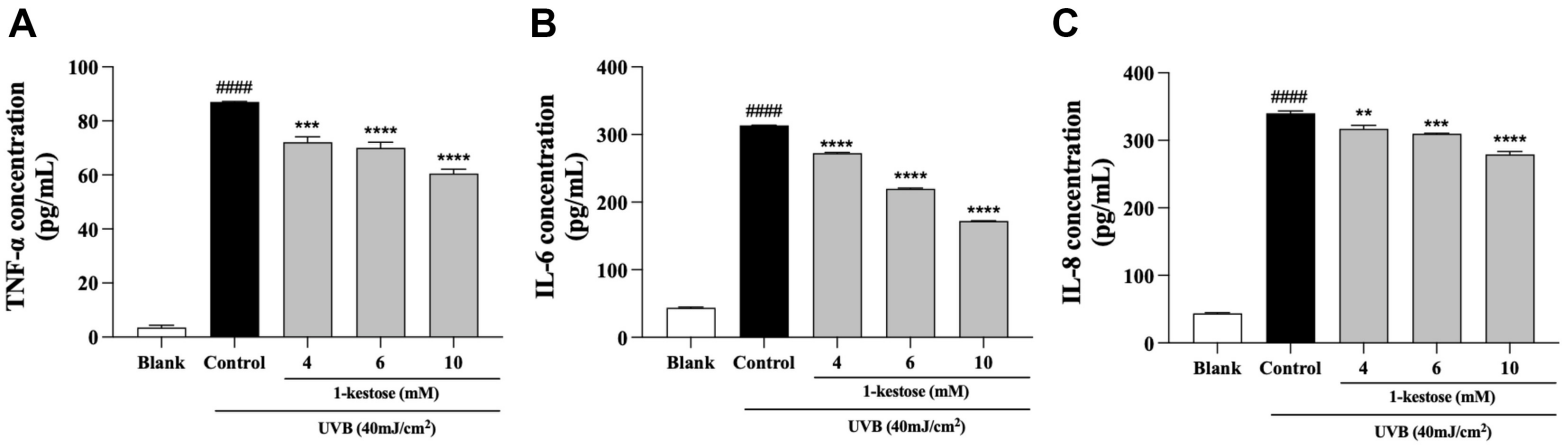
Effect of 1-kestose pretreatment on the secretion of UVB radiation-induced pro-inflammatory in HaCaT cells. The secretion of (**A, B, C**) TNF-α, IL-6 and IL-8 secretion was measured using ELISA kit, followed by treatment with 1-kestose (4, 6, and 10 mM) for 12 h. All data are expressed as mean ± SD (*n* = 3). #### *p* < .0001 vs. blank group, ** *p* < .01, *** *p* < .001, and **** *p* < .0001 vs control group.

To evaluate the effect of 1-kestose on TNF-α, IL-6, and IL-8 secretion in UVB radiation-induced HaCaT cells, the levels of TNF-α, IL-6, and IL-8 in the supernatants were performed by ELISA kits. As shown in Fig. 2, exposure of HaCaT cells to UVB increased the TNF-α, IL-6, and IL-8 expression compared with the control group. Treatment with 1-kestose obviously reduced cytokines secretion. 10 mM 1-kestose decreased UVB radiation-induced TNF-α, IL-6, and IL-8 cytokine levels by 69.6%, 54.9%, and 82.1%, respectively.

### 1-Kestose Downregulated UVB Radiation-Induced Expression of MMP-1, MMP-3 and Upregulated Procollagen Type I Synthesis

**Fig. 3:**
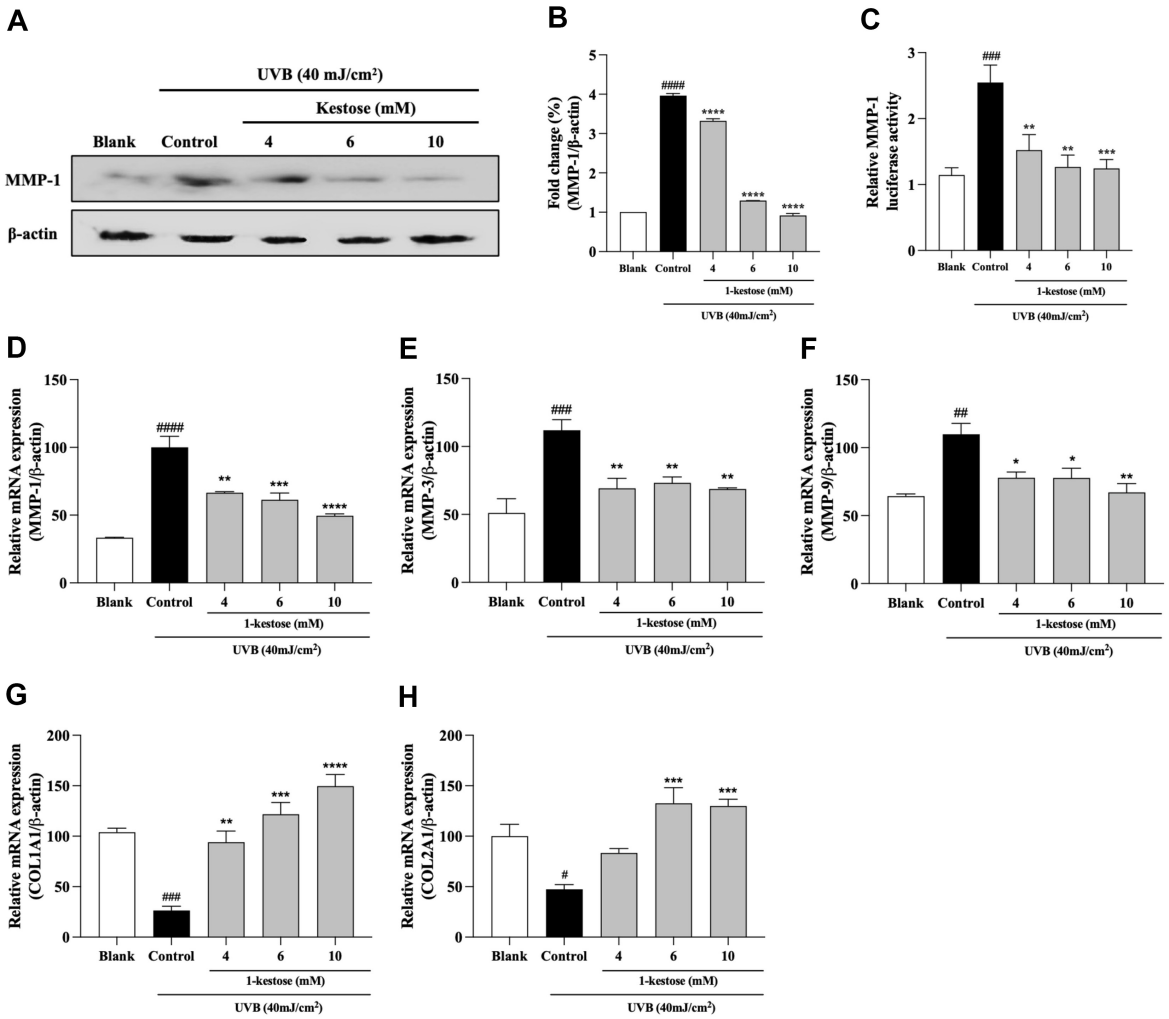
1-kestose suppresses MMPs and Type I procollagen expression in UVB radiation-induced HaCaT cells. (**A, B**) Level of expressed MMP-1 was detected by western blotting 12 h after UVB irradiation. Band intensity was quantified by ImageJ and normalized to β-actin. (**C**) Dual-luciferase reporter assay was evaluated to measure the MMP-1 promoter activity in the HaCaT cells. The mRNA expression of (**D**) MMP-1, (**E**) MMP-3, (**F**) MMP-9, (**G**) COL1A1 and (**H**) COL2A1 was measured using RT-PCR, followed by treatment with 1-kestose (4, 6, and 10 mM) for 12 h. All data are expressed as mean ± SD (*n* = 3). # *p* < .05, ## *p* < .01, ### *p* < .001, and #### *p* < .0001 vs. blank group, * *p* < .05, ** *p* < .01, *** *p* < .001, and **** *p* < .0001 vs control group.

The main causes of skin photoaging were indicated to be MMPs secretion and suppression in procollagen synthesis. To determine the possible role of 1-kestose in MMP-1 expression in radiated UVB in HaCaT cells, we first confirmed whether MMP-1 expression is downregulated by UVB irradiation. As shown in Fig. 3A and B, MMP-1 expression level was significantly increased by UVB exposure. However, the expression of MMP-1 was decreased by 1-kestose in a dose-dependent manner by 16.2%, 67.3%, and 87.7%, respectively. Similarly, UVB exposure enhanced UVB-induced MMP-1 promoter activity, whereas treatment with 1-kestose remarkably suppressed MMP-1 promoter activity (Fig. 3C). To confirm the gene expression levels related to the regulation of MMPs and type genes of procollagen expression, MMP-1, -3, -9, COL1A1 and COL2A1 mRNA levels were measured by RT-PCR. As shown in Fig. 3D–H, the expression levels of MMP-1, -3, and -9 from control group were significantly elevated, whereas expression levels of COL1A1 and COL2A1 were significantly decreased compared with blank group. However, this change was significantly advanced by pretreatment with 1-kestose. 10 mM 1-kestose decreased expression levels of MMP-1, -3, and -9 by 50.3%, 31.3%, and 32.6%, respectively and elevated expression levels of COL1A1 and COL2A1 by 466.4% and 173.6%, respectively. These results suggest that 1-kestose decreases the expression of MMP-1 and regulates the expression of MMPs and type I collagen at the transcriptional level.

### 1-Kestose Inhibits AP-1 Activation and Regulates UVB Radiation-Induced MAPKs Phosphorylation in HaCaT Cells

**Fig. 4:**
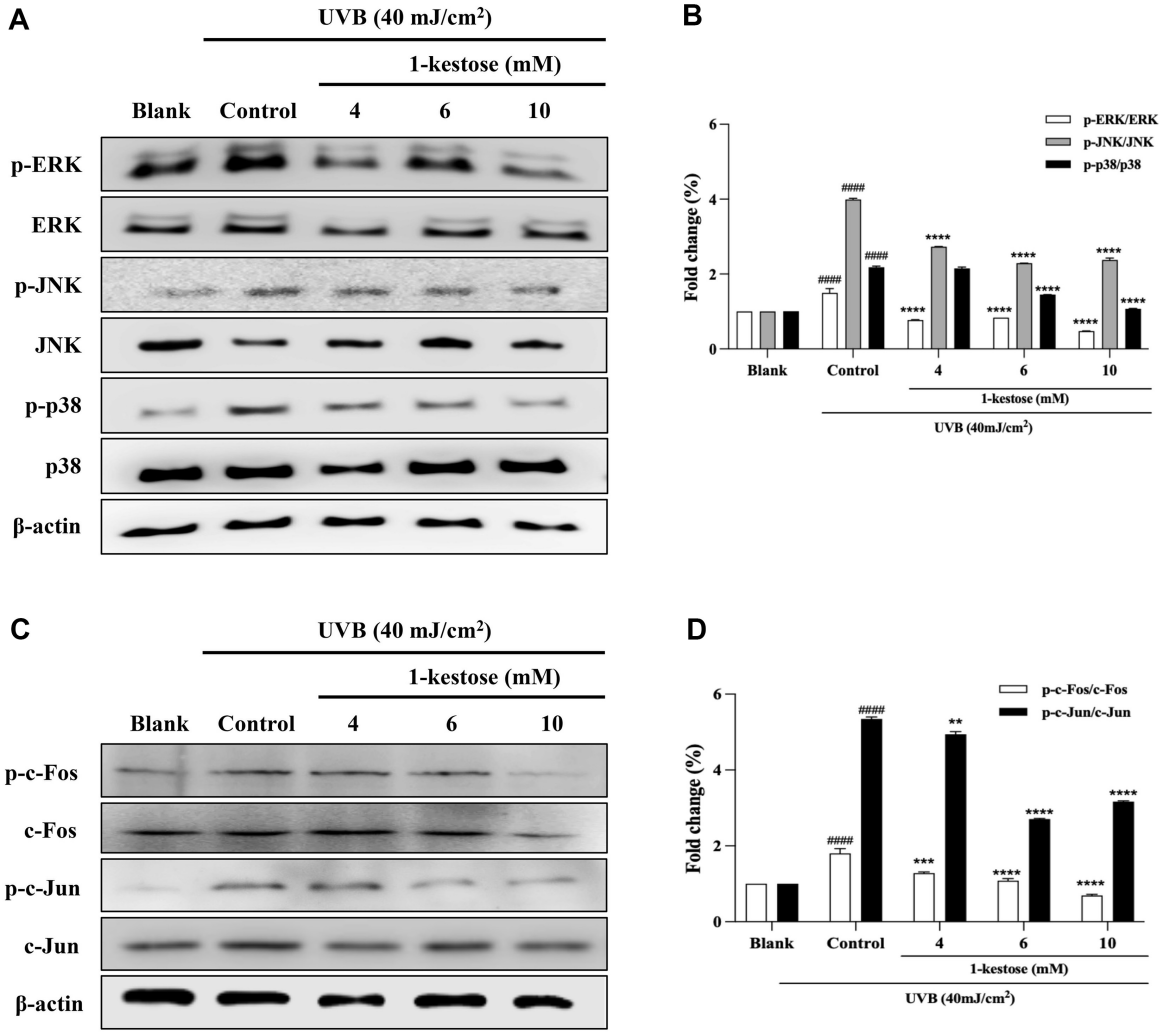
1-kestose blocked MAPKs, AP-1 pathway in UVB radiation-induced HaCaT cells. (**A, B, C, D**) Total cell lysates were performed to assess the expression for MAPKs and AP-1 by Western blotting analysis, followed by treatment with 1-kestose (4, 6, and 10 mM) for 12 h. Band intensity was quantified by ImageJ and normalized to β-actin. All data are expressed as mean ± SD (*n* = 3). # *p* < .05 and #### *p* < .0001 vs. blank group, ** *p* < .01 and **** *p* < .0001 vs control group.

Activation of AP-1 expression stimulates the secretion of MMPs that degrade collagen [20]. Thus, we further investigated the effect of 1-kestose on AP-1 expression in UVB radiation-induced HaCaT cells. UVB irradiation showed secretion of c-Jun and c-Fos phosphorylation elevated by western blotting. Conversely, 1-kestose pretreatment effectively decreased the secretion of p-c-Jun and p-c-Fos 61.5% and 40.7% at 10 mM, respectively (Fig. 4A and B). AP-1/MAPKs signal are enhanced by UVB-induced excessive reactive oxygen species production. As shown in Fig. 4C and D, UVB-induced HaCaT cells noticeably decreased levels of p-ERK, p-JNK, and p-p38, whereas 10 mM 1-kestose pretreatment suppressed UVB-induced p-ERK, p-JNK, and p-p38 expression in a dose-dependent manner by 32.2%, 41.4%, and 40.4%, respectively. These results demonstrate that 1-kestose suppresses MMP-1 expression by inhibiting the phosphorylation AP-1/MAPKs, which play important roles in MMP-1 expression.

### 1-Kestose Inhibits NF-κB Activation in HaCaT Cells

**Fig. 5:**
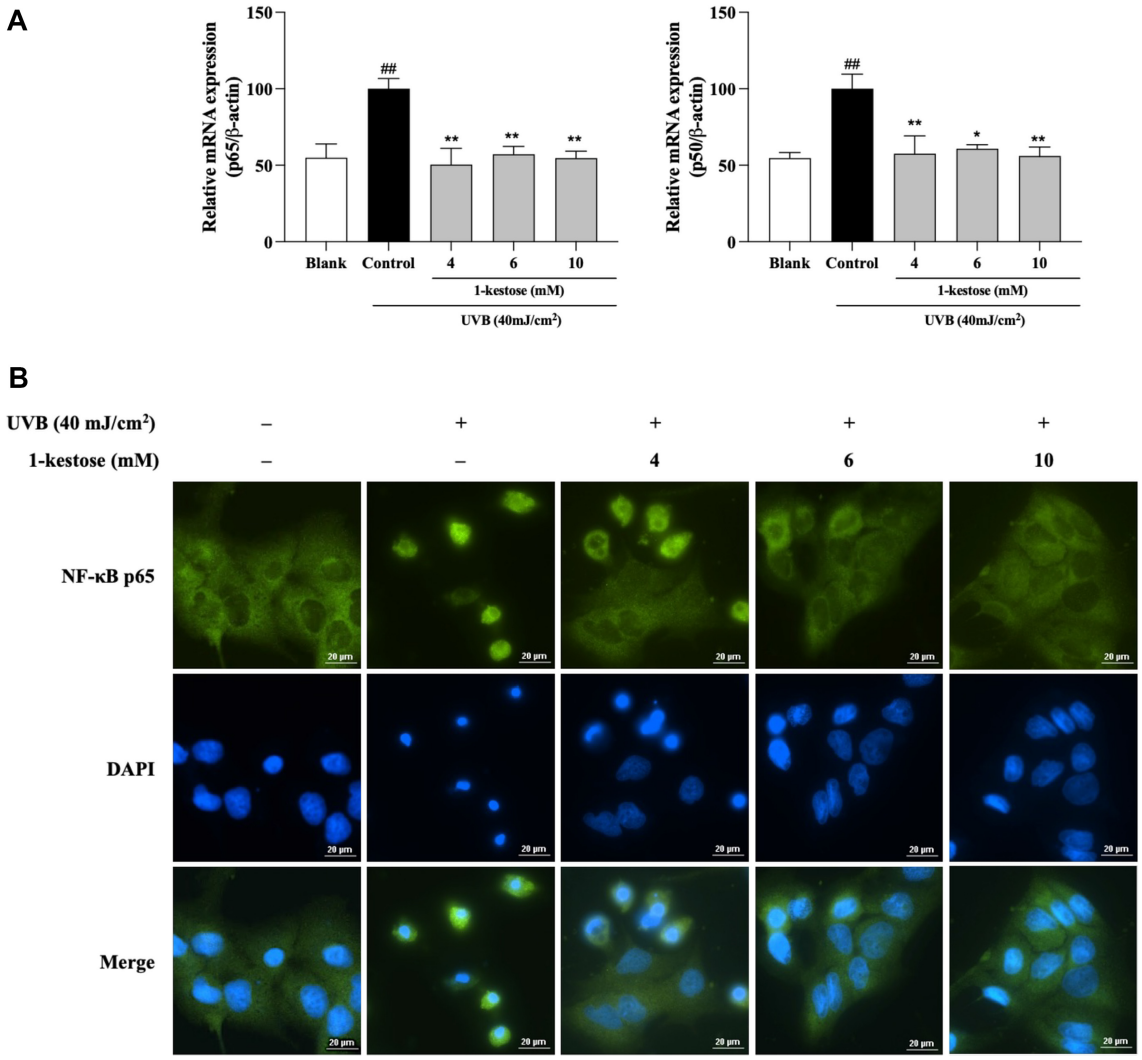
1-kestose inhibits NF-κB translocation in UVB radiation-induced HaCaT cells. (**A, B**) The mRNA expression of NF-κB p65 and p50 was measured by RT-PCR, followed by treatment with 1-kestose (4, 6, and 10 mM) for 12 h. (**C**) Nuclear translocation of NF-κB p65 was determined by immunofluorescence by an overlay of green p65 staining with DAPI staining. ## *p* < .01 vs. blank group, * *p* < .05 and ** *p* < .01, vs control group.

To clarify the effect of 1-kestose on UVB radiation-induced activation of NF-κB, we assessed the NF-κB mRNA expression level and p65 translocation analysis. As shown in Fig. 5A, control group was increased NF-κB (p65 and p50) expression. 1-kestose pretreatment inhibited NF-κB expression in a dose-independent manner. Furthermore, p65 nuclear translocation showed same results that 1-kestose can inhibited p65 nuclear translocation in UVB-induced HaCaT cells (Fig. 5B).

### 1-Kestose Up-Regulated TGF-β1/Smads Pathway

**Fig. 6:**
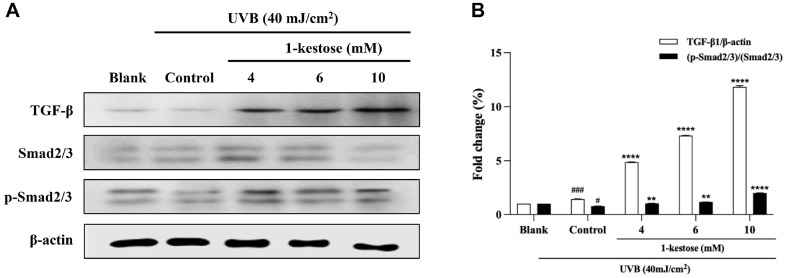
1-kestose improve TGF-β1/Smad signaling pathway in UVB radiation-induced HaCaT cells. HaCaT cells were irradiated with UVB, followed by treatment with 1-kestose (4, 6 and 10 mM) for 12 h. (**A, B**) Total cell lysates were performed to assess the expression for TGF-β1/Smad by Western blotting analysis. Band intensity was quantified by ImageJ and normalized to β-actin. All data are expressed as mean ± SD (*n* = 3). # *p* < .05 and ### *p* < .001 vs. blank group, **** *p* < .0001 vs control group.

As shown in Fig. 6A, 6B, and control group significantly inhibited TGF-β1 and phosphorylation of Smad2/3 expression. However, 1-kestose pretreatment improved these effects by 713.7% and by 149.3% compared to control at 10 mM 1-kestose concentration.

## Discussion

Photoaging is defined as the characteristic changes to skin exposed to UV radiation [21]. Excessive exposure to ultraviolet radiation leads to a significant increase in skin damage, characterized by prominent signs of photoaging such as wrinkle formation and skin roughness, attributed to cytokines secreted by keratinocytes [2]. In the present study, 1-kestose can provide photoprotective functions by preventing the UVB-induced decrease in HaCaT cell viability and altering various photoaging processes.

ROS generation is a significant cause of skin photodamage. Intracellular ROS generation by extracellular stimulation induces DNA oxidative damage and is closely related to pro-inflammatory generation [22]. In our present study, the level of ROS production was analyzed using DCFH-DA, and UVB irradiation increased ROS level while 1-kestose pretreatment decreased ROS production and suppressed secretion of pro-inflammatory cytokines. Previous our study have shown that 1-kestose alleviates atopic dermatitis against Th2-associated inflammation [16]. In addition, 1-kestose is used as a functional ingredient in products, such as food and cosmetics due to its potential antioxidant properties [23, 24]. These results support that 1-kestose reduces inflammation and protects the skin barrier. Based on these results, the hypothesis was formulated that 1-kestose has a high potential to prevent skin photoaging.

During photoaging, collagen and elastin fibers are able to be degraded by UVB-induced MMPs [25]. Therefore, inhibiting UVB-induced MMPs and collagenase expressions may play critical roles in preventing photoaging. MMP-1 can be capable of degrading the most abundant Type I collagen. Furthermore, MMP-3 activates MMP-1 and degrades Type IV collagen in the basement membrane, while collaborating with MMP-9 to degrade collagen fragments [26]. In this study, 1-kestose can decrease the expression levels of MMP-1, MMP-3, and MMP-9 and increased COL1A1 and COL2A1. UVB radiation-induced high secretion of MMPs is complex and regulates many signaling including MAPK/AP-1 complex/NF-κB and TGF-β/Smad [8, 27].

MAPKs including ERK, JNK and p38 are the major signaling molecules in response to UV irradiation and regulate cell proliferation, differentiation, apoptosis, and inflammatory response, and MMPs secretion [20]. The AP-1 transcription factor, which is involved in the expression of MMPs, has been reported to be regulated by MAPKs [28]. Besides, ROS accumulation by UVB irradiation stimulates the phosphorylation of MAPKs, and c-jun dimerizes with c-fos, and AP-1 complex to activate gene expression of MMPs [29]. Like AP-1, transcription factor NF-κB is a central mediator of inflammation-related heterodimer consisting of p65 and p50 subunits and is concerned with regulating the MMP-1, -3, and -9 genes expression in photoaging. Here, we confirmed that 1-kestose inhibits the UVB-induced phosphorylation expression of MAPKs, AP-1, and NF-κB. Therefore, we inferred that in the presence of UVB-induced inflammatory conditions, 1-kestose could inhibit MMP-1 expression and collagen degradation through MAPKs, AP-1, and NF-κB inhibition.

Type I collagen is the central structural protein in skin connective tissue [30]. Synthesis of procollagen initiates binding of TGF-β1 to the receptor and stimulates the phosphorylated Smad2/3 complex [12, 27]. It has been reported that UV radiation impairs the TGF-β1/Smad pathway and reduces the expression of type I procollagen [31]. In this study, 1-kestose up-regulated expression levels of TGF-β1 and p-Smad2/3 in UVB-induced impaired cells. These results indicate that 1-kestose prevent photoaging activity through TGF-β1/Smad pathway.

## Conclusion

In summary, this study demonstrated that 1-kestose decreased UVB radiation-induced ROS production, thereby suppressing expression of MMPs and pro-inflammatory. In addition, 1-kestose inhibited the MAPKs/AP-1/NF-κB signal, which effectively avoids degradation of Type I procollagen gene expression. Finally, 1-kestose was shown to have a photoprotective effect by the TGF-β1/Smad signaling pathway. These findings suggest that 1-kestose may serve as a skin protectant against UVB-induced inflammation and photoaging. Therefore, this study suggests that 1-kestose will provide a pharmacological basis for future studies on reducing inflammation and preventing photoaging.

## Figures and Tables

**Table 1:** The primer sequences for quantitative PCR.

List	Forward (5’-3’)	Reverse (5’-3’)
MMP-1	CACAGCTTCCCAGCGACTC	GTCCCGATGATCTCCCCTGA
MMP-3	ATCCTACTGTTGCTGTGCGT	CATCACCTCCAGAGTGTCGG
MMP-9	ATCCAGTTTGGTGTCGCGGAGC	GAAGGGGAAGACGCACAGCT
COL1A1	CAGGTACCATGACCGAGACG	AGCACCATCATTTCCACGAG
COL2A1	GCAACGTGGTGAGAGAGGAT	CCTGTCGTCCGGGTTCAC
NF-κBp50	CACAAGGCAGCAAATAGACG	GAGTTAGCAGTGAGGCACCA
NF-κBp65	CAGGCGAGAGGAGCACAGATAC	TCCTTTCCTACAAGCTCGTGGG
β-actin	AGCGAGCATCCCCCAAAGTT	GGGCACGAAGGCTCATCATT
